# Editorial: Immunopeptidomic Approaches for the Identification of Tumor (neo)Antigens

**DOI:** 10.3389/fimmu.2022.923776

**Published:** 2022-05-11

**Authors:** Jennie R. Lill, Michal Bassani-Sternberg

**Affiliations:** ^1^Department of Microchemistry, Proteomics, Lipidomics and Next Generation Sequencing, Genentech Inc., South San Francisco, CA, United States; ^2^Ludwig Institute for Cancer Research, Lausanne, Agora Research Center, Lausanne, Switzerland

**Keywords:** immunopeptidome, mass spectrometry, proteomics, cancer immunology, major histocompability complex

The field of immunopeptidomics has evolved over the past twenty years since researchers required ample cellular material and manual curation of mass spectrometry spectra to discern the sequence of MHC I and II peptides (1–3). With the evolution of highly sensitive and accurate mass-spectrometers and next generation sequencing techniques such as RNAseq and Exome sequencing, proteogenomics has allowed the customization of cell/sample specific reference databases. It therefore enabled the detection of mutated neo-antigens, covering single nucleotide variant somatic mutations, translocation events and other genetic aberrations in the context of MHC I and II immunopeptidomes (4, 5). In this Research Topic about Immunopeptidomic Approaches for the Identification of Tumor (neo)Antigens, each of the manuscripts combine genomic and/or proteomic analyses to fully characterize the immunopeptidome. A multi-omic approach was employed by Fang et al. to identify novel tumor specific antigens from thymic carcinomas. Thymic carcinomas are aggressive epithelial neoplasms resulting from various genetic events including microsatellite instability, genome doubling and gene fusions. By interrogating the genome and transcriptome of thymic carcinoma cancer patients they revealed that frameshift insertions and deletions in KMT2C and CYLD frequently produce neoantigens. In addition, several fusion derived neoantigens were predicted across patients. Their findings have allowed more insight into the molecular mechanisms behind this disease and also a potential route to immunotherapeutic intervention.

An integrated genomic, proteomic and immunopeptidomic approach was also employed by Olsson et al. in one of two studies exploring the effect of cellular treatment with IFN-gamma on the immunopeptidome. Using their suite of tools they assessed differential expression of full length proteins upon IFN gamma treatment in a human lymphoma cell line and demonstrated that a modest proteomic response to this cytokine could create large changes in the cells epitope repertoire. They found that these changes were also largely reflected at the immunopeptidome level. To fully dissect the data they developed a classification system that allowed one to distinguish between peptides which are differentially presented due to changes in antigen processing versus differential expression at the protein level.

Goncalves et al. also interrogated the role of IFN gamma and how it modulates the immunopeptidome of triple negative breast cancer (TNBC) cells to identify potential T cell targets for development anti-cancer vaccines. Again, taking a multi-omics approach, the team showed that treatment with this cytokine resulted in significant modulation, with minimal overlap between untreated and treated cells across the HLA-I immunopeptidome. In addition, MHC II expression was only observed on treated cells, thereby providing another mechanism for T cell mediated cell death *via* the Helper T cell response. T

In concert, the work presented by Olsson et al. and Goncalves et al. demonstrate the high degree of plasticity in the immunopeptidome following cytokine stimulation. Understanding how both IFN gamma is playing this role *in vivo* in a tumor, or *via* endogenous stimulation in a therapeutic setting could allow one to develop both improved in silico algorithms for predicting MHC I and II presentation or develop strategies by which immunopeptide repertoires are intentionally reshaped to improve endogenous or vaccine-induced anti-tumor immune responses and potentially anti-viral immune responses.

Along these lines Marino et al. also employed a proteogenomics approach to assess the biogenesis of MHC I and II peptide presentation of activated immune cells. Antigen presenting cells (APC) play a critical role in cancer immunotherapy as they are key mediators of T cell activation and resultant tumorigenic cell death. The antigen presenting machinery subtly differs between APC type and here, how those differences translate into peptide signatures such as peptide length was explored.

Marino et al. characterized naturally presented HLA-I and -II immunopeptidomes from autologous immune cells having distinct functional and biological states including CD14+ monocytes, immature DC (ImmDC) and mature DC (MaDC) monocyte-derived DCs and naive or activated T and B cells. Significantly longer HLA peptides were identified upon activation in a HLA allotype specific across these cell types. This was apparent in the self-peptidome upon cell activation and in the context of presentation of exogenously loaded antigens, suggesting that peptide length is an important feature. Better understanding these molecular mechanisms, as also described for cytokine treatment, has potential implications on the rational design of anti-cancer vaccines.

The majority of work presented in this Research Topic is associated with characterizing peptides derived from canonical source proteins. However, over the past decade, more attention has been focused on the non-canonical and “dark matter” proteome. In the review by Minati et al. a “roadmap” towards defining actionable tumor specific antigens is described. Here, they highlight the potential sources of tumor specific antigens (TSAs) that have been derived from non-canonical open reading frames, and discuss the potential implications of these as targets for development of immunotherapy. They hypothesize that in line with the inter- and intra-tumor heterogeneity, discrete families of TSAs may be enriched in different cancer types.

To catalogue this data, Lu et al. describe an open-access database to provide a systematic resource for storage and query of the validated neoantigens in cancer research. In this special addition they introduce version 2.0 of dbPepNeo 0 (http://119.3.70.71/dbPepNeo2/home.html) where they provide > 800 high confidence neoantigens > 80,000 low confidence HLA peptidomes for data mining. Their manuscript introduces two algorithms, DeepCNN-Ineo, which predicts the immunogenicity of MHC I peptides and BLASTdb which performs sequence similarity searches against data resources stored in dbPepNeo2.0. This tool has a web based interface for easy data mining and is a valuable resource to the field for Neo-antigen information storage.

Overall the manuscripts within this Research Topic demonstrate that the field of immunopeptidomics is evolving, providing us with clearer insight into how we can better predict MHC I and II presentation in various cellular contexts, with the vision that these new “rules” can help provide the community with better cancer immunotherapeutic tools in the near future.

## Author Contributions

BS and JL have reviewed and edited the manuscripts and have composed this editorial summarizing key take home messages for this special edition of Frontiers in Immunology focusing on Immunopeptidomic Approaches for the Identification of Tumor (neo)Antigens. All authors contributed to the article and approved the submitted version.

## Conflict of Interest

Author JL is employed by Genentech, a member of the Roche Organization.

The remaining author declares that the research was conducted in the absence of any commercial or financial relationships that could be construed as a potential conflict of interest.’

## Publisher’s Note

All claims expressed in this article are solely those of the authors and do not necessarily represent those of their affiliated organizations, or those of the publisher, the editors and the reviewers. Any product that may be evaluated in this article, or claim that may be made by its manufacturer, is not guaranteed or endorsed by the publisher.
